# ALG-bFGF Hydrogel Inhibiting Autophagy Contributes to Protection of Blood–Spinal Cord Barrier Integrity *via* PI3K/Akt/FOXO1/KLF4 Pathway After SCI

**DOI:** 10.3389/fphar.2022.828896

**Published:** 2022-03-07

**Authors:** Renkan Zhang, Ling Xie, Fangfang Wu, Ji Xu, Leilei Lu, Lin Cao, Lei Li, Weiyang Meng, Hongyu Zhang, Chuxiao Shao, Xiaokun Li, Daqing Chen

**Affiliations:** ^1^ Department of Emergency, The Second Affiliated Hospital and Yuying Children’s Hospital, Wenzhou Medical University, Wenzhou, China; ^2^ School of Pharmaceutical Sciences, Wenzhou Medical University, Wenzhou, China; ^3^ Department of Hepatopancreatobiliary Surgery, Lishui Central Hospital, The Fifth Affiliated Hospital of Wenzhou Medical University, Lishui Hospital of Zhejiang University, Lishui, China

**Keywords:** bFGF, sodium alginate hydrogel, spinal cord injury (SCI), autophagy, blood–spinal cord barrier (BSCB), PI3K/Akt/FOXO1/KLF4

## Abstract

Promoting blood–spinal cord barrier (BSCB) repair at the early stage plays a crucial role in treatment of spinal cord injury (SCI). Excessive activation of autophagy can prevent recovery of BSCB after SCI. Basic fibroblast growth factor (bFGF) has been shown to promote BSCB repair and locomotor function recovery in SCI. However, the therapeutic effect of bFGF *via* direct administration on SCI is limited because of its rapid degradation and dilution at injury site. Based on these considerations, controlled release of bFGF in the lesion area is becoming an attractive strategy for SCI repair. At present, we have designed a sustained-release system of bFGF (called ALG-bFGF) using sodium alginate hydrogel, which is able to load large amounts of bFGF and suitable for *in situ* administration of bFGF *in vivo*. Here, traumatic SCI mice models and oxygen glucose deprivation (OGD)–stimulated human brain microvascular endothelial cells were performed to explore the effects and the underlying mechanisms of ALG-bFGF in promoting SCI repair. After a single *in situ* injection of ALG-bFGF hydrogel into the injured spinal cord, sustained release of bFGF from ALG hydrogel distinctly prevented BSCB destruction and improved motor functional recovery in mice after SCI, which showed better therapeutic effect than those in mice treated with bFGF solution or ALG. Evidences have demonstrated that autophagy is involved in maintaining BSCB integrity and functional restoration in animals after SCI. In this study, SCI/OGD exposure–induced significant upregulations of autophagy activation-related proteins (Beclin1, ATG5, LC3II/I) were distinctly decreased by ALG-bFGF hydrogel near the baseline and not less than it both *in vivo* and *in vitro*, and this inhibitory effect contributed to prevent BSCB destruction. Finally, PI3K inhibitor LY294002 and KLF4 inhibitor NSC-664704 were applied to further explore the underlying mechanism by which ALG-bFGF attenuated autophagy activation to alleviate BSCB destruction after SCI. The results further indicated that ALG-bFGF hydrogel maintaining BSCB integrity by inhibiting autophagy activation was regulated by PI3K/Akt/FOXO1/KLF4 pathway. In summary, our current study revealed a novel mechanism by which ALG-bFGF hydrogel improves BSCB and motor function recovery after SCI, providing an effective therapeutic strategy for SCI repair.

## Introduction

Spinal cord injury (SCI) is a major cause of permanent disability (Chen et al., 2013; Witiw and Fehlings, 2015). Blood–spinal cord barrier (BSCB) plays a protective role in the spinal parenchyma by preventing entry of toxic circulating molecules, immune cells, and inflammatory substances into the spinal cord (Bartanusz et al., 2011). BSCB integrity is primarily determined by junction complexes containing tight junctions (TJ) and adherent junctions (AJ) (Stamatovic et al., 2016). Upon injury, the tight junctions formed by nonfenestrated endothelial cells are lost, leading to BSCB breakdown and increase of BSCB permeability (Tran et al., 2018). Previous studies have shown that traumatic SCI-induced BSCB disruption is involved in SCI pathophysiological processes, such as spinal cord edema and secondary nerve injury. Disruption of BSCB leads to infiltration of proinflammatory cells to the injured area, which then produces proinflammatory cytokines and neurotoxic products that impair neuronal and synaptic function and further lead to death of glial cells and neurons, aggravating neurological deficits (Hawkins and Davis, 2005; Zlokovic, 2008). More recently, it has been reported that disruption of BSCB is associated with increased mortality while improving BSCB function can significantly reduce secondary nerve injury (Winkler et al., 2014). Therefore, promoting BSCB recovery at the early stage plays a crucial role in treatment of SCI.

Autophagy, also known as macroautophagy, is an essential lysosome-dependent intracellular degradation pathway that wraps long-lived proteins and damaged organelles into a double-membrane vesicle and then transports them to lysosome for degradation (Shaui Zhang et al., 2018). Autophagy plays an important role in maintaining cellular homeostasis and recycling proteins and organelles (Mizushima and Komatsu, 2011). Basal level of autophagy ensures the physiological clearance of damaged cellular components, which is beneficial to cell growth, development, and differentiation (Fortini et al., 2016; Jennifer et al., 2019; Gross and Graef, 2020). However, excessive autophagy can lead to cellular dysfunction and autophagic death of cells. Ample evidence has indicated that autophagy is involved in maintenance of BSCB integrity after SCI (Zhou, et al., 2016a; Zhou et al., 2016b; Zhou et al., 2017; Wang et al., 2018; Tong et al., 2018). Moreover, inhibition of autophagy activation is beneficial to reduce BSCB permeability at the early stage of SCI (Wang et al., 2018). Therefore, autophagy is an important mechanism for regulation of BSCB and for SCI recovery.

Basic fibroblast growth factor (bFGF), a member of the FGF family, plays a large number of important roles in proliferation, morphogenesis, and inhibition of apoptosis during development through a complex signal transduction system (Wang et al., 2012; Zhang et al., 2013a; Wang et al., 2015). bFGF has been proved to protect the integrity of BSCB by reducing the loss of AJ and TJ proteins after SCI (Ye et al., 2016). In addition, there is a study indicating that bFGF promotes SCI recovery through inhibition of excessive autophagy (Zhang et al., 2013a). Thus, we hypothesize that inhibition of autophagy by bFGF contributes to maintenance of BSCB integrity after SCI. Previous studies have revealed that autophagy is regulated by the PI3K/Akt signaling pathway (Lin et al., 2011; Zhang et al., 2012), and PI3K/Akt/FOXO1 pathway is involved in pathological processes of SCI and cerebral hemorrhage (Li et al., 2020; Xu et al., 2021). It has also been reported that PI3K/Akt/FOXO1 pathway participates in the regulation of autophagy (Liu et al., 2014). KLF4 has been proved to be the downstream target of FOXO1 (Yusuf et al., 2008) and involved in regulation of autophagy in a variety of disease conditions (Liao et al., 2015; Liu et al., 2015; Xue et al., 2020). Therefore, we hypothesize that bFGF-mediated downregulation of autophagy is important for alleviating BSCB disruption and improving locomotor functional recovery by PI3K/Akt/FOXO1/KLF4 signaling pathway after SCI. However, as a biological macromolecular protein, systemic administration of bFGF for SCI treatment has significant shortcomings including short half-life, difficulty of passing through the BSCB, and tumorigenicity in healthy tissues because of its mitogenic potential (Li et al., 2011). To overcome these shortcomings, *in situ* delivery systems have been designed to allow local diffusion of bFGF in the lesion area of spinal cord. Therefore, hydrogel and other biomaterials loading growth factors (GFs) have been well developed and widely applied for SCI (Wang et al., 2017; Wang et al., 2019; Albashari et al., 2020; Hu et al., 2020).

Biomaterials or bioscaffold have been proved to show great prospect in nerve injury repair (Chen et al., 2020; Qian et al., 2020). Sodium alginate hydrogel is a biocompatible, non-toxic, and non-immunogenicity polymer formed by the combination of G region of sodium alginate and divalent cation (Giri et al., 2012). This hydrogel has several advantages of 1) high affinity for various GFs, 2) sustained release of these GFs in a stable manner, 3) protection of GFs from enzymatic hydrolysis, and 4) avoiding the side effects of high concentrations of GFs at the injection site. It has been widely used as a scaffold material for tissue engineering and cell transplantation (Li et al., 2019; Rastogi and Kandasubramanian, 2019; Zhang and Zhao, 2020). After SCI, a large amount of divalent calcium ions accumulated in the injured area (Young et al., 1982; Young and Koreh, 1986; Singh et al., 2019), and provided a natural gelatinizing environment for sodium alginate. A previous study has shown that sodium alginate hydrogel-loaded FGF21 has a good therapeutic effect in SCI (Zhu et al., 2021). To improve the therapeutic efficacy of bFGF in SCI, sodium alginate hydrogel was applied in the study. This sodium alginate–loaded bFGF (ALG-bFGF) hydrogel can not only reduce the frequency of administration of bFGF but also maintain bFGF release for a long enough time to sustain the treatment of SCI.

In this study, we postulated that sustained release of bFGF from ALG-bFGF hydrogel significantly improves locomotor functional recovery and alleviates BSCB disruption. Furthermore, we hypothesized that the role of ALG-bFGF hydrogel in protecting BSCB integrity is mediated by the inhibition of autophagy. Using SCI mice models, oxygen glucose deprivation (OGD)–stimulated human brain microvascular endothelial cells (HBMECs), and specific inhibitors, the work demonstrates that ALG-bFGF hydrogel inhibits autophagy activation by regulation of the PI3K/Akt/FOXO1/KLF4 pathway, which contributes to restoration of BSCB integrity and locomotor function recovery after SCI.

## Materials and Methods

### Preparation of ALG-bFGF Hydrogel

Sodium alginate dry powder and bFGF dry powder were dissolved together in tri-distilled water to prepare different concentrations (1, 5, 10 mg/ml) of sodium alginate hydrogel containing 0.48 mg/ml bFGF. Following magnetic stirring for 2 h and sterilization with 0.22 μm filtration membrane, the sodium alginate–bFGF (ALG-bFGF) mixture was stored at 4°C for future use.

### Micromorphology Assessment of ALG and ALG-bFGF Hydrogels

The micromorphology of dehydrated ALG and ALG-bFGF hydrogels was detected using scanning electron microscopy (SEM). Shortly, the ALG or ALG-bFGF mixture was frozen with liquid nitrogen after being dried in a freeze dryer for 24 h. Whereafter, they were carefully crosscut and placed into the conductive glue for gold plating. Finally, the morphological structures of ALG and ALG-bFGF were observed by SEM.

### SCI Model and Treatment

Healthy adult female C57BL/6N mice (weight 20–25 g) were purchased from the Laboratory Animal Center of Wenzhou Medical University and housed in a standard condition at 23 ± 2°C maintaining 12/12 h light–dark cycle and had free access to water and food for at least 7 days before exposure to SCI surgery. Mice were randomly arranged to five groups: sham, SCI, SCI + ALG, SCI + bFGF, and SCI + ALG-bFGF. Traumatic SCI was performed in mice as previously described. In short, mice were anesthetized with 4% (w/v) chloral hydrate (10 ml/kg, i.p.), and then a T10 laminectomy was performed to expose the spinal cord. Subsequently, a 10-g weight was dropped from a height of 1.5 cm onto the exposed spinal cord to induce a moderate SCI contusion. Dissected muscle, fascia, and skin were then sutured layer by layer with 4-0 absorbable lines. After injury, mice were further housed in standard condition and received bladder emptying manually twice daily until bladder reflex was established. Mice in the sham group had received only spinal cord exposure but without injury. All animal use and care protocols conformed to the guidelines of Laboratory Animal of China National Institutes of Health. All animal experimental protocols were approved by the Animal Care and Use Committee of Wenzhou Medical University.

Same volume (5 µl) of ALG hydrogel, free bFGF solution (0.48 mg/ml), or ALG-bFGF hydrogel was injected *in situ* into the injured spinal cord using a microsyringe. To investigate the role of inhibition of autophagy by ALG-bFGF hydrogel in maintaining BSCB integrity in mice that suffered from SCI, an autophagy specific inhibitor 3-methyladenine (3-MA, 15 mg/kg) or an autophagy activator rapamycin (RAPA, 0.5 mg/kg) was intraperitoneally injected after SCI. To explore the underlying molecular mechanism of ALG-bFGF in SCI repair, a PI3K specific inhibitor LY294002 (50 nmol/kg) was injected *in situ* into the injured spinal cord by a microsyringe. An equivalent volume of saline was administrated to mice in SCI control group.

### Evans Blue Dye Assays

Evans blue (EB) is the most widely used tracer to quantify BSCB integrity. Albumin-bound Evans blue cannot pass through the normal BSCB but can permeate and stain tissue blue when BSCB is disrupted, thus EB presence in spinal cord tissue indicates alteration in BSCB leakage. Mice were intravenously injected with 2% EB dye (0.25 ml) *via* tail vein at 3 days after SCI. Two hours later, mice were anesthetized and sacrificed by intracardiac perfusion with 0.9% saline, and then the injured spinal cord tissues were obtained. The EB dye in the spinal cord was extracted with formamide and the supernatant was then incubated at 72°C for 3 days following centrifugation (1,500×*g*) for 20 min at room temperature (RT). The quantity of EB extravasation was examined at an excitation wavelength of 610 nm and an emission wavelength of 680 nm by a spectrophotometer. Finally, the quantity of EB obtained from spinal cord was determined according to a standard curve.

### Locomotion Recovery Assessment

The BMS score and footprint test were used to evaluate locomotion recovery of mice’s hind limbs at 0, 1, 3, 5, 7, 14, 21, and 28 days after surgery. The BMS scores range from 0 to 9; higher scores indicate better restoration of locomotion. The mice were placed individually on an open field and allowed to move freely for 5 min, and the locomotor ability of the mice was evaluated by BMS score based on scoring tail posture, paw posture, trunk stability, coordination, and posterior ankle joint mobility. The footprint test was applied by dipping the mice’s fore limbs in blue dye and hind limbs in red dye; the mice walked freely on a white runway, and the trajectories of these movements were recorded.

### Cell Culture and Treatment

The HBMECs were cultured in endothelial cell medium (ECM) containing 5% fetal bovine serum (FBS), 1% penicillin/streptomycin mixture, and 1% endothelial cell growth supplement at 37°C with a humidified atmosphere containing 5% CO_2_. The HBMECs and all of the reagents were purchased from ScienCell Research Laboratories.

To uncover the underlying mechanism of ALG-bFGF in promoting BSCB integrity *in vitro*, the OGD-induced HBMEC model was established to simulate injured microenvironment after SCI. For OGD-stimulated HBMEC model, cells were incubated in FBS-free ECM for 6 h in an anaerobic chamber in which the oxygen level remained less than 0.5%. After OGD treatment, HBMECs were further incubated for 12 h under normal culture conditions. Cells were treated with 50 ng/ml free bFGF or ALG-bFGF for 2 h before exposure to OGD treatment. To evaluate the effect of ALG-bFGF on autophagy in OGD-stimulated HBMEC, cells were pre-treated with ALG-bFGF combined with RAPA (100 nM) or 3-MA (5 μM) for 2 h before OGD stimulation. To further investigate whether ALG-bFGF maintains BSCB integrity after SCI is partly regulated by PI3K/Akt/FOXO1/KLF4 pathway *in vitro*, PI3K specific inhibitor LY294002 or KLF4 specific inhibitor NSC-664704 was applied in OGD-stimulated HBMECs. Cells were pre-treated with LY294002 (20 μM) for 2 h and with NSC-664704 (10 μM) for 6 h before exposure to OGD intervention, respectively.

### Western Blot Analysis

Tissues and cells were lysed with RIPA lysis buffer supplemented with 1% protein phosphatase inhibitor mixture, and the sample was then centrifuged (12,000 rpm) at 4°C for 15 min to get the protein supernatant. The concentration of total protein was detected using the BCA assay kit (Thermo). After separation in a 10–12% SDS-PAGE gel, the proteins were then transferred onto a PVDF membrane (Bio-Rad). The membrane was blocked for 1.5 h with 5% (w/v) non-fat milk dissolved in tris-buffer saline solution containing 0.1% (v/v) Tween-20 (TBST) at RT and further incubated overnight at 4°C with the following primary antibodies: ZO-1 (1:1,000), P120-catenin (1:1,000), β-catenin (1:1,000), occludin (1:1,000), claudin-5 (1:1,000), ATG5 (1:1,000), Beclin1 (1:1,000), AKT (1:1,000), p-AKT (1:1,000), FOXO1 (1:1,000), KLF4 (1:1,000), β-actin (1:10,000), and LC3II (1:1,000; Abcam). Next, after washing three times with TBST, the membrane was submerged in a corresponding HRP-conjugated secondary antibody for 1 h at RT. Signals of protein expression were collected by a ChemiDoc XRS imaging system (Bio-Rad). The relative density of the band was quantified by ImageJ software. All of the primary antibodies (except for anti-LC3II) were purchased from Proteintech.

### Immunofluorescence Staining

Spinal cord tissues were fixed in 4% paraformaldehyde (PFA) dissolved in PBS for 24 h. Next, the samples were embedded in paraffin and then cut into 5-µm-thick slices. The slices were successively deparaffinized and rehydrated. For immunofluorescence staining in HBMECs, cells’ climbing slices were also fixed with 4% PFA for 15 min. After washing with PBS, the tissue or cell slices were blocked with 5% BSA dissolved in PBS at 37°C for 30 min. Subsequently, the slices were incubated with the following antibodies at 4°C overnight: ZO-1 (1:250), β-catenin (1:250), LC3 (1:500), and CD31 (1:500; Santa Cruz). Then, the slices were further treated with the corresponding Alexa Fluor conjugated secondary antibody (1:1,000; Abcam) for 1 h at 37°C. DAPI was used for nuclei staining. Fluorescence intensity in cells was imaged using a Leica microscope.

### Statistical Analysis

All results are displayed as the mean ± SEM. Significant differences between two groups were determined using Student’s *t*-test. When more than two groups were compared, statistical significance was analyzed using one-way ANOVA and Dunnett’s *post hoc* test. A *p*-value <0.05 was considered statistically significant.

## Results

### ALG-bFGF Hydrogel Characterization and Treatment for SCI

**FIGURE 1:**
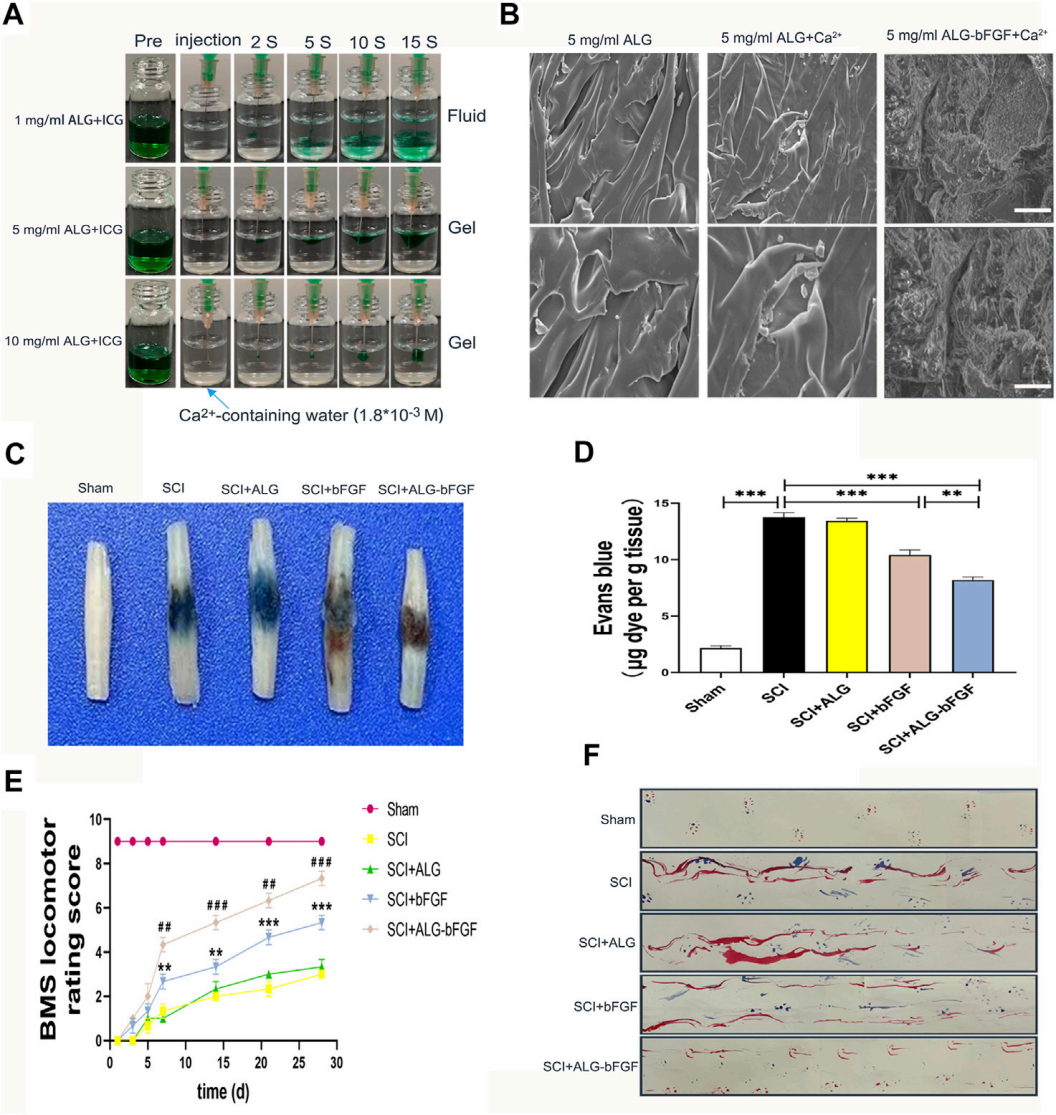
Characterization of ALG-bFGF hydrogel improves SCI repair. **(A)** Gelation process of hydrogels with different concentrations of sodium alginate. **(B)** SEM images and shape of hydrogels in different groups. Scale bar = 20 μm, and scale bar = 10 μm (enlarged figure). **(C)** Assessment of BSCB integrity by detecting permeability of Evans blue dye into the injured spinal cord tissue of mice at 3 days after SCI. **(D)** Quantitative analysis of Evans blue dye of spinal cord (μg/g) in each group. **(E)** Statistical analysis of BMS scores of mice from different groups at the indicated time point after SCI. **(F)** Footprint test of mice in different groups at 28 days after SCI. All data are displayed as the mean ± SEM, *n* = 3. **p < 0.01, ***p < 0.001. ##*p* < 0.01, ###*p* < 0.001 *vs*. the SCI + bFGF group.

We first prepared and determined different concentrations of sodium alginate solution at which it will become colloid, and the mixture of ALG solution and indocyanine green (ICG, an indicator) was injected into CaCl_2_ solution. Results in Figure 1A showed that the mixture with 1 mg/ml ALG diffused within 15 s into CaCl_2_ solution while it did not diffuse when ALG solution is 5 mg/ml and 10 mg/ml, indicating that sodium alginate solution at 5 mg/ml and 10 mg/ml was gelatinized immediately in CaCl_2_ solution. However, the ALG hydrogel at 10 mg/ml with high viscosity is not suitable for injection *in situ* into the spinal cord; therefore, ALG hydrogel at 5 mg/ml was selected for subsequent experiments. The morphology of ALG hydrogel at 5 mg/ml and ALG-loaded bFGF (ALG-bFGF) hydrogel was also observed under SEM. ALG hydrogel showed a 3D structure with smooth surface, but the ALG-bFGF hydrogel had a fibrous porous structure, where the bFGF protein can be adsorbed (Figure 1B). Collectively, the fibrous porous structure of ALG at 5 mg/ml is suitable for loading bFGF and injection *in situ* for drug delivery.

This study next examined whether ALG-bFGF protects BSCB against damage and promotes motor functional recovery in mice after SCI. To evaluate the effects of ALG-bFGF hydrogel on mitigating BSCB damage induced by traumatic SCI, EB dye was used to detect BSCB permeability at 3 days after SCI. Traumatic SCI resulted in a significant increase of EB dye exosmosis compared with that in sham group, indicating that the BSCB integrity was damaged after SCI. The level of EB dye extravasation was not changed after treatment of ALG hydrogel but was significantly decreased both in bFGF and ALG-bFGF administration groups, and the level of EB in ALG-bFGF group was less than that in bFGF-treated group (Figures 1C,D). BMS score and footprint analysis were further performed to assess whether ALF-bFGF improves motor recovery in mice after SCI. Results in Figure 1E showed that traumatic SCI caused the BMS score in mice to sharply decrease, and there were no significant improvements in mice treated with ALG hydrogel, bFGF solution, and ALG-bFGF hydrogel at early stage (1, 3, 5 days after injury). However, the bFGF and ALG-bFGF group showed a significant positive effect at 7, 14, 21, and 28 days after injury. Moreover, ALG-bFGF group showed a better locomotion outcome than that in bFGF group. Consistently, results of footprint analysis at 28 days after injury also displayed the most restoration of hind leg locomotion in ALG-bFGF group (Figure 1F). Taken together, ALG-bFGF hydrogel injected *in situ* into the spinal cord could more effectively promote locomotor functional recovery in mice following SCI.

### ALG-bFGF Hydrogel Reduces BSCB Damage by Inhibiting Activation of Autophagy

**FIGURE 2:**
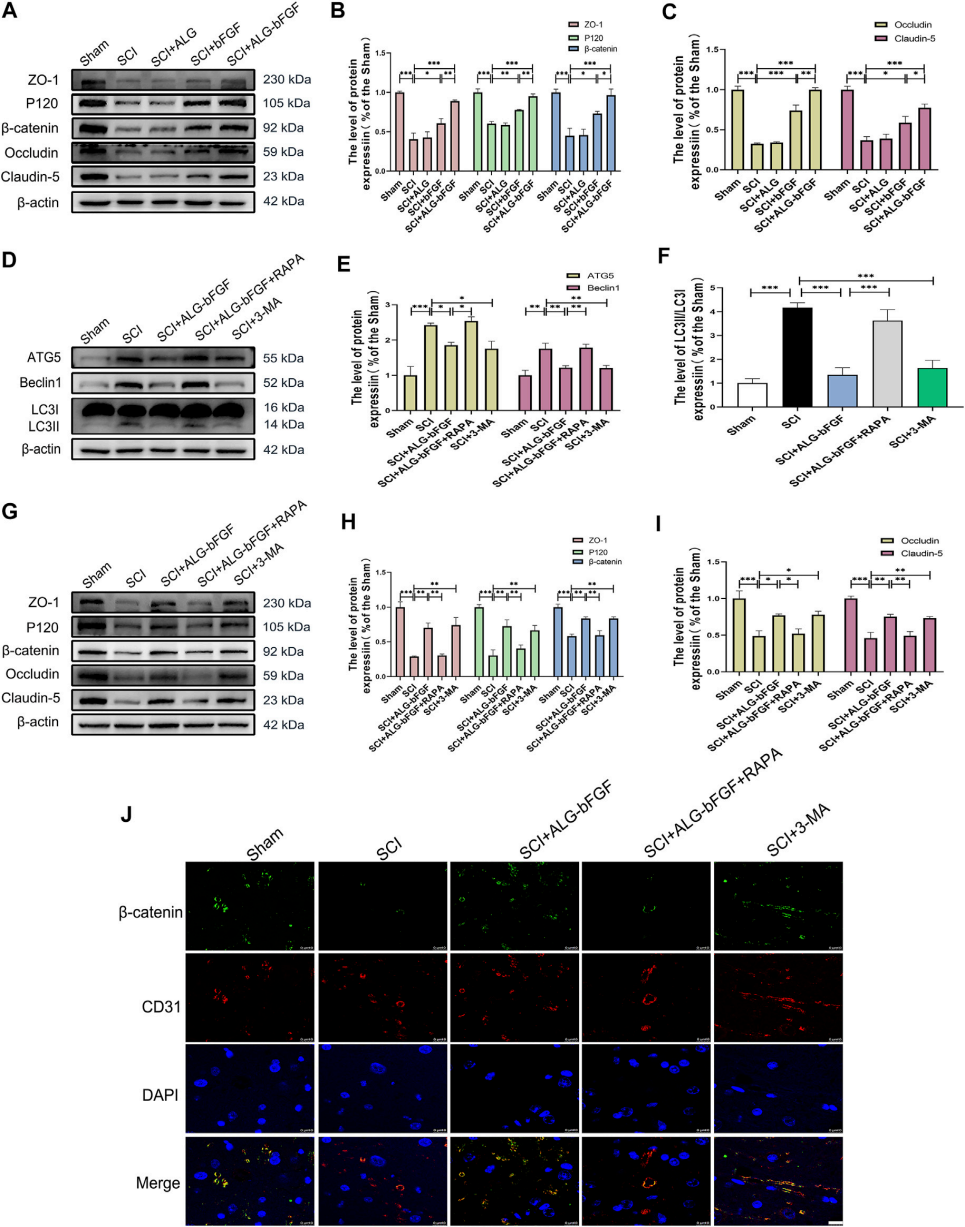
ALG-bFGF hydrogel upregulates the expressions of junction proteins *via* inhibiting autophagy activation at 3 days after SCI. **(A)** Western blot image showing the protein expressions of TJ proteins (ZO-1, claudin-5, occludin) and AJ proteins (β-catenin, P120) in spinal cord of mice from different groups. **(B,C)** Quantitative analysis of the protein levels showed in **(A)**. **(D)** Western blot image showing the protein expression of ATG5, Beclin1, and LC3 in spinal cord of mice from different groups. **(E,F)** Quantitative analysis of protein expressions of ATG5, Beclin1, and LC3II/I presented in **(D)**. **(G)** Western blot image showing the expression of TJ proteins and AJ proteins in spinal cord of mice that suffered from different interventions. **(H,I)** The quantitative analysis of the data presented in **(G)**. **(J)** Representative immunofluorescence stain of β-catenin (green) and CD31 (red) in spinal cord from different groups at 3 days after injury. Scale bar = 10 µm. All results are showed as the mean ± SEM, *n* = 3. *p < 0.05, **p < 0.01, and ***p < 0.001.

To further confirm the effects of ALG-bFGF hydrogel on protecting BSCB against destruction induced by SCI, the protein levels of tight junction (TJ) and adhesion junction (AJ) proteins were determined by Western blot. As shown in Figures 2A–C, both of the protein levels of AJ (P120 and β-catenin) and TJ (ZO-1, claudin-5, and occludin) were distinctly reduced at 3 days after injury, while these reductions were dramatically attenuated by bFGF and ALG-bFGF hydrogel treatment, and there was a better attenuation in ALG-bFGF hydrogel group than that in bFGF group. The result indicates that ALG-bFGF effectively alleviates BSCB damage by attenuating AJ and TJ protein loss induced by SCI.

Evidence indicates that SCI activates autophagy and inhibition of autophagy contributes to maintain BSCB integrity and improve movement outcomes after SCI. To indicate the underlying mechanism by which ALG-bFGF prevents BSCB destruction after SCI, the expression levels of autophagy activation–related proteins were determined by Western blot. Results in Figures 2D–F showed that protein expressions of autophagy activation markers (ATG5, Beclin1, and LC3Ⅱ/LC3Ⅰ ratio) were sharply increased after SCI while they significantly decreased with the treatment of ALG-bFGF or an autophagy inhibitor 3-methyladenine (3-MA), and the inhibitory effect of ALG-bFGF on autophagy was reversed by rapamycin (RAPA, an inducer of autophagy activation). These results suggest that ALG-bFGF hydrogel attenuates SCI-induced activation of autophagy.

To further investigate whether inhibiting autophagy activation by ALG-bFGF hydrogel is beneficial for maintaining BSCB integrity, 33-MA and rapamycin were administered in mice after SCI, and then the protein levels of AJ and TJ were detected by Western blot and immunofluorescence staining at 3 days after injury. The result exhibited that ALG-bFGF hydrogel or 3-MA attenuated SCI-induced loss of TJ proteins (ZO-1, occludin, and claudin-5) and AJ proteins (β-catenin and P120), while this inhibitory effect was overturned by administration of rapamycin (Figures 2G–I). Consistently, the fluorescence intensity of β-catenin in endothelial cells (labeled with CD31) was decreased after SCI, but enhanced after treatment of ALG-bFGF hydrogel, which was reversed by administration of rapamycin (Figure 2J). Taken together, similar to the role of 3-MA, these data suggest that ALG-bFGF hydrogel inhibiting autophagy activation may play an important role in protection of BSCB integrity in mice after SCI.

### ALG-bFGF Inhibits Autophagy Activation and Reduces the Loss of AJ and TJ Proteins in OGD-Stimulated HBMECs

**FIGURE 3:**
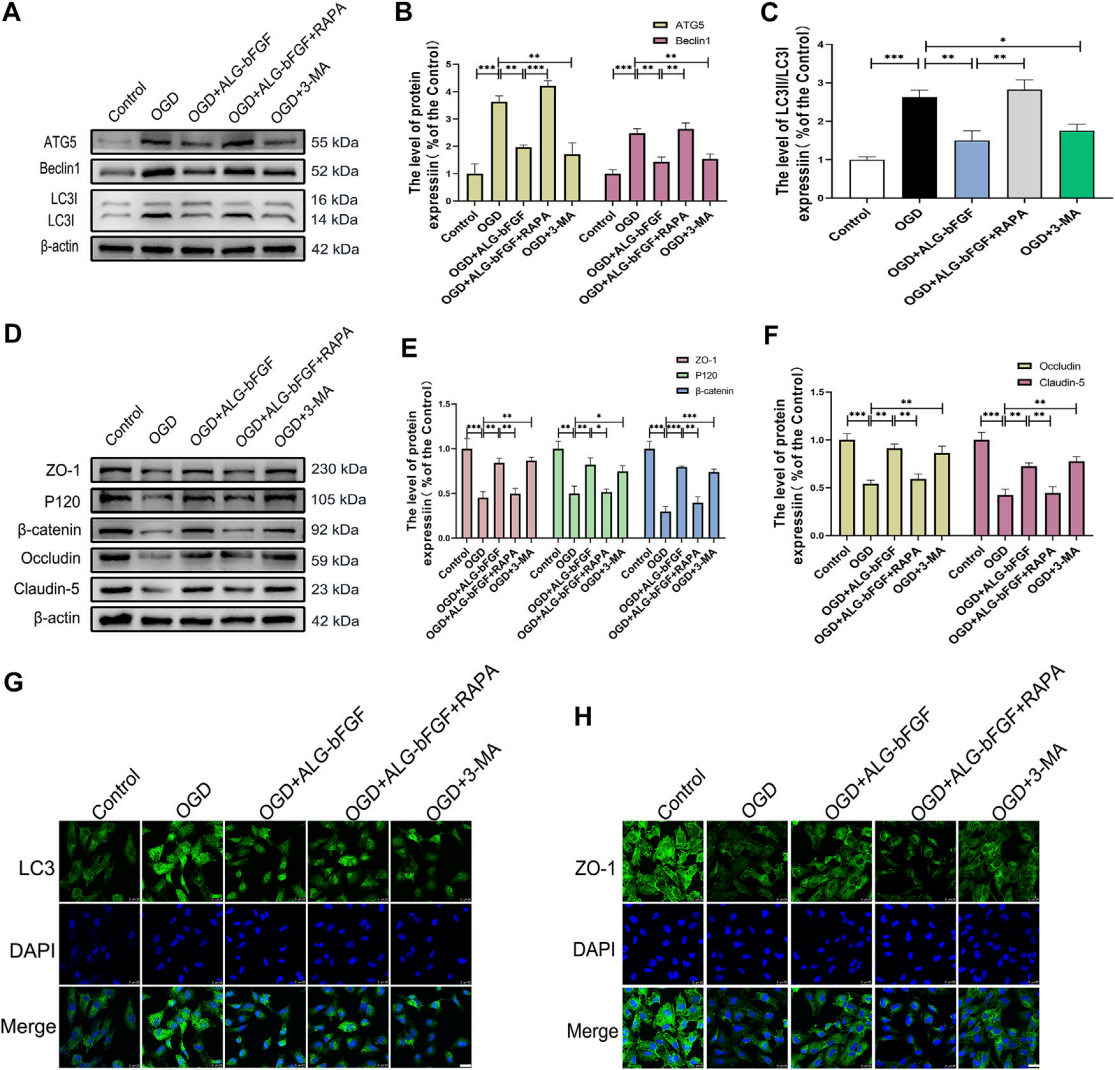
ALG-bFGF hydrogel reduces the loss of junction proteins by inhibiting autophagy in OGD-stimulated HBMECs. **(A)** Western blot image showing the protein expression of autophagy markers (ATG5, Beclin1, LC3) in HBMECs that suffered from different treatments. **(B,C)** The quantitative analysis of protein expression of ATG5, Beclin1, and LC3II/I showed in **(A)**. **(D)** Western blot image showing the proteins expression of ZO-1, occludin, claudin-5, P120, and β-catenin in HBMECs exposed to different conditions. **(E,F)** Statistical analysis of Western blot data presented in **(D)**. **(G,H)** Immunofluorescence stain of LC3 and ZO-1 in HBMECs exposed to different conditions, respectively. Scale bar = 25 µm. All results are displayed as the mean ± SEM, *n* = 3. *p < 0.05, **p < 0.01, ***p < 0.01.

To further verify if inhibition of autophagy activation by ALG-bFGF hydrogel is involved in maintaining BSCB integrity, a HBMEC injury model was stimulated by OGD, and then protein levels of BSCB integrity indicator (AJ and TJ proteins) were determined after incubation with ALG-bFGF hydrogel combined with or without rapamycin. As expected, the results displayed that expressions of autophagy-related proteins were significantly increased after OGD exposure while they decreased with administration of ALG-bFGF hydrogel or 3-MA. Also, the inhibitory effect of ALG-bFGF hydrogel was distinctly reversed by rapamycin (Figures 3A–C), indicating that OGD-induced activation of autophagy was attenuated by ALG-bFGF hydrogel in HBMECs. Conversely, the protein expressions of AJ (β-catenin and P120) and TJ (ZO-1, occludin, and claudin-5) were distinctly downregulated in HBMECs that suffered from OGD but dramatically upregulated when incubated with ALG-bFGF hydrogel or 3-MA. Also, this inductive effect of ALG-bFGF hydrogel was overturned by rapamycin (Figures 3D–F), suggesting that ALG-bFGF hydrogel reducing loss of AJ and TJ may be regulated by inhibition of autophagy activation. Consistently, fluorescence intensity of LC3 was diminished, but fluorescence intensity of ZO-1 was enhanced in HBMECs incubated with ALG-bFGF hydrogel compared with OGD-treated group, which was reversed by administration of rapamycin (Figures 3G,H). Collectively, these data strengthen that attenuation of autophagy activation by ALG-bFGF hydrogel contributes to maintain BSCB integrity after SCI.

### PI3K/AKT/FOXO1/KLF4 Pathway Participates in ALG-bFGF Hydrogel Maintaining BSCB Integrity

**FIGURE 4:**
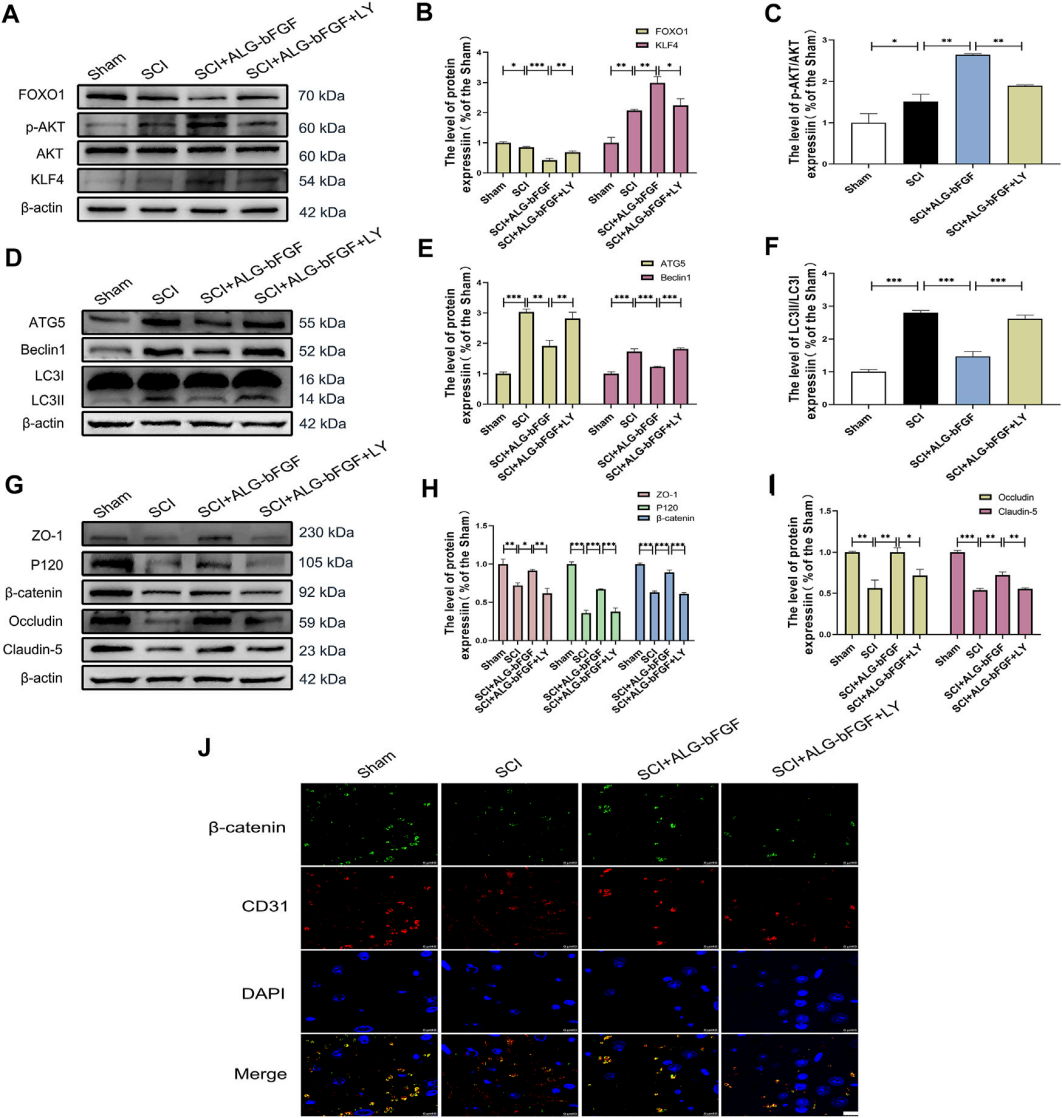
PI3K inhibitor LY294002 attenuates the effects of ALG-bFGF hydrogel on autophagy and loss of BSCB-related proteins at 3 days after SCI. **(A)** Western blot image showing the protein expressions of FOXO1, KLF4, p-AKT, and AKT in damaged spinal cord of different groups. **(B,C)** Quantification of the data displayed in **(A)**. **(D)** Western blot image showing the expression of autophagy indicators, ATG5, Beclin1, and LC3 in spinal cord of mice from different groups. **(E,F)** Statistical analysis of the data showed in **(D)**. **(G)** Western blot image of ZO-1, occludin, claudin-5, P120, and β-catenin in spinal cord tissue of mice from different groups. **(H,I)** Quantification of protein expressions displayed in **(G)**. **(J)** Representative immunofluorescence stain of β-catenin (green) and CD31 (red) in spinal cord of mice from different groups. Scale bar = 10 µm. All results are showed as the mean ± SEM, *n* = 3. *p < 0.05, **p < 0.01, and ***p < 0.001.

To explore whether PI3K/AKT/FOXO1/KLF4 participates in the protective effect of ALG-bFGF hydrogel on BSCB integrity, LY294002, a PI3K inhibitor, was performed in mice treated with ALG-bFGF hydrogel after SCI. As shown in Figures 4A–C exhibiting protein expressions of AKT, FOXO1, KLF4, and phosphorylation level of AKT, ALG-bFGF hydrogel significantly increased the p-Akt/AKT ratio, decreased FOXO1, and upregulated KLF4 expression compared with that in mice after SCI, which were distinctly reversed by administration of LY294002. In addition, the effect of PI3K/AKT/FOXO1/KLF4 pathway on autophagy activation and prevention of BSCB destruction after SCI was next examined. LY294002 successfully overturned the inhibitory effect of ALG-bFGF hydrogel on autophagy after SCI (Figures 4D–F), indicating that PI3K/AKT/FOXO1/KLF4 pathway may be involved in ALG-bFGF hydrogel-mediated inhibition of autophagy activation after SCI. Meanwhile, the protective role of ALG-bFGF hydrogel in the prevention of TJ and AJ protein loss caused by SCI was sharply diminished by LY294002 treatment (Figures 4G–I). Consistent with the result, the fluorescence intensity of β-catenin in endothelial cells (established by CD31) was reduced in mice after traumatic SCI but highlighted by treatment of ALG-bFGF hydrogel, which was weakened by administration of LY294002 (Figure 4J). Collectively, these data indicate that the protective effect of ALG-bFGF hydrogel on BSCB integrity *via* inhibiting autophagy activation is partly regulated by PI3K/AKT/FOXO1/KLF4 pathway after SCI.

### PI3K Inhibitor Attenuates Effects of ALG-bFGF Hydrogel on Inhibition of Autophagy Activation and Upregulation of AJ and TJ Proteins *In Vitro*

**FIGURE 5:**
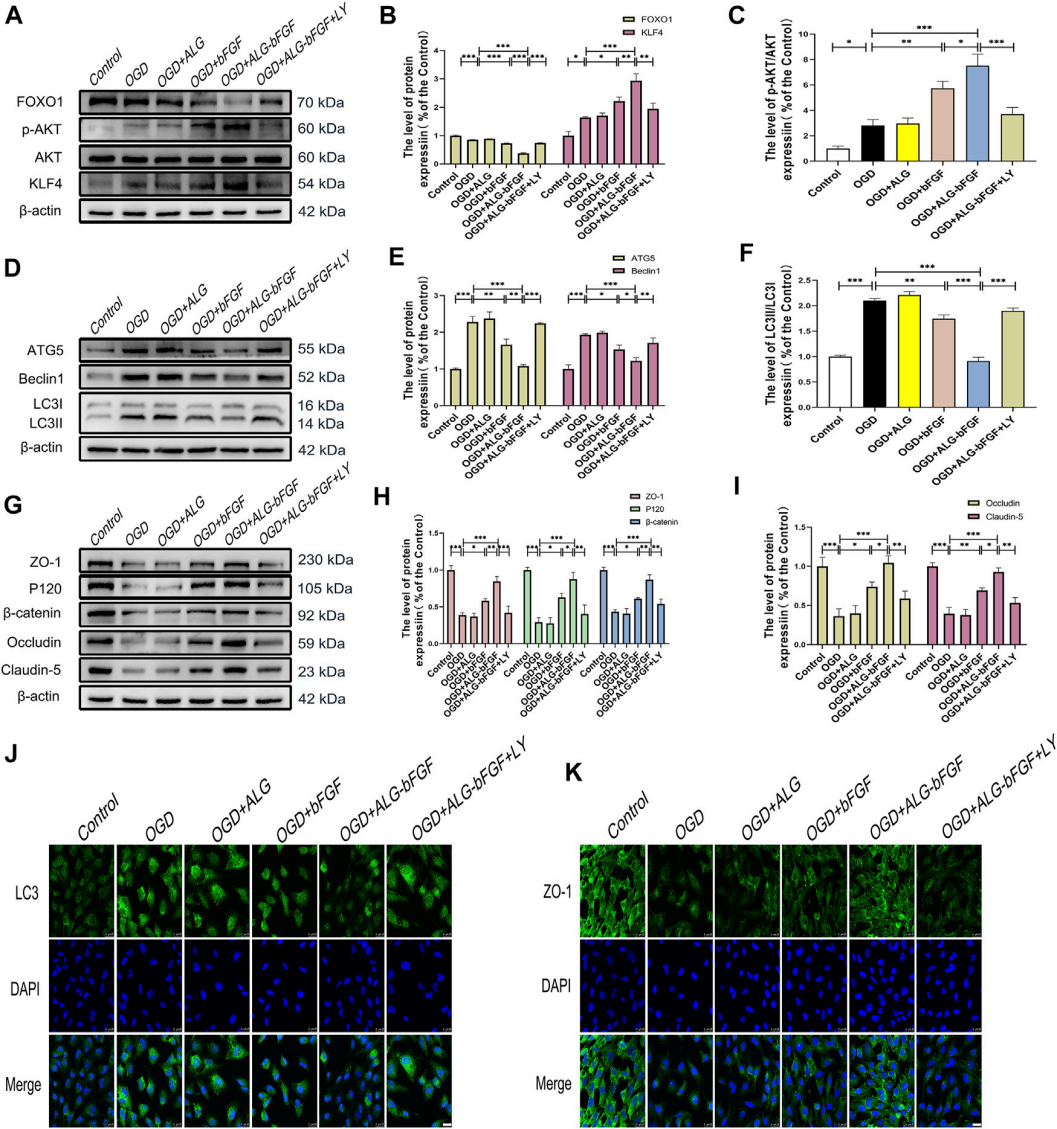
PI3K inhibitor LY294002 reverses the effects of ALG-bFGF hydrogel on autophagy and loss of BSCB-related proteins in OGD-induced HBMECs. **(A)** Western blot image exhibiting the protein expressions of FOXO1, KLF4, p-AKT, and AKT in HBMECs from different groups. **(B,C)** Quantification of the protein expressions presented in **(A)**. **(D)** Western blot image showing the expression of ATG5, Beclin1, and LC3 in HBMECs that suffered from different interventions. **(E,F)** Quantitative analysis of the protein expressions displayed in **(D)**. **(G)** Western blot exhibiting the expression of ZO-1, occludin, claudin-5, P120, and β-catenin in HBMECs exposed to different conditions. **(H,I)** Quantitative analysis of protein expressions showed in **(G)**. **(J,K)** Representative immunofluorescence stain of LC3 and ZO-1 in HBMECs suffered from different intervention, respectively. Scale bar = 25 µm. All results are displayed as the mean ± SEM, *n* = 3. *p < 0.05, **p < 0.01, and ***p < 0.001.

To further affirm ALG-bFGF hydrogel inhibiting autophagy activation and reducing AJ and TJ protein loss is regulated through PI3K/AKT/FOXO1/KLF4 pathway, LY294002 was applied in OGD-induced HBMECs, and protein levels of autophagy-related proteins and indicators of BSCB integrity were determined using Western blot analysis. Consistent with results from mice that suffered from SCI, PI3K inhibitor LY294002 significantly overturned the effects of ALG-bFGF hydrogel on protein expressions of FOXO1 as well as KLF4 and on the p-Akt/AKT ratio in OGD-induced HBMECs (Figures 5A–C). These results consolidate that ALG-bFGF hydrogel plays a protective role in SCI partly through regulation of PI3K/AKT/FOXO1/KLF4 pathway. In addition, the ALG-bFGF hydrogel attenuating autophagy activation was also dramatically reversed by LY294002 in OGD-injured HBMECs (Figures 5D–F), which is consistent with the result of fluorescence intensity of LC3 showed in Figure 5J. These data strengthen the evidence that PI3K/AKT/FOXO1/KLF4 pathway participates in ALG-bFGF hydrogel inhibiting activation of autophagy in mice after SCI. In the meantime, the AJ and TJ protein levels were apparently reduced in HBMECs after OGD exposure while they remarkably increased by incubation of ALG-bFGF hydrogel, which was distinctly overturned by LY294002 (Figures 5G–I). These results were verified by immunofluorescence staining of ZO-1 in OGD-induced HBMECs (Figure 5K), touching on the fact that ALG-bFGF hydrogel preventing BSCB disruption is partly regulated by the PI3K/AKT/FOXO1/KLF4 pathway after SCI. Collectively, these data combined with the results from Figure 3 support that ALG-bFGF hydrogel inhibiting autophagy activation to prevent BSCB damage is regulated by PI3K/AKT/FOXO1/KLF4 pathway after SCI.

### KLF4 Participates in Effects of ALG-bFGF Hydrogel on Autophagy and AJ and TJ Protein Expression in OGD-Treated HBMECs

**FIGURE 6:**
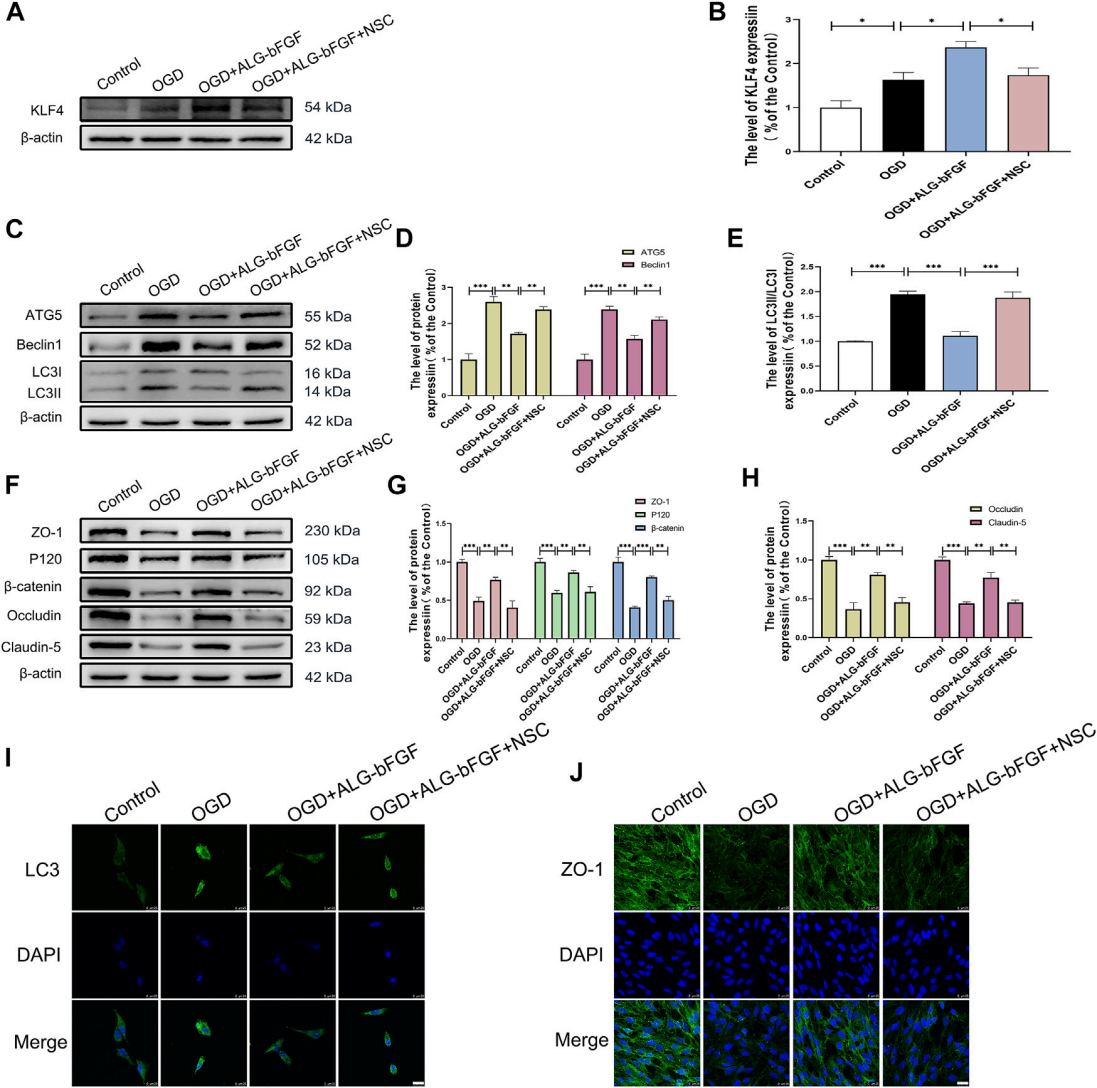
KLF4 inhibitor NSC-664704 attenuates the effects of ALG-bFGF hydrogel on autophagy and loss of BSCB-related proteins in OGD-induced HBMECs. **(A)** Western blot image showing ALG-bFGF hydrogel-induced upregulation of KLF4 is attenuated by NSC-664704 in OGD-stimulated HBMECs. **(B)** Quantitative analysis of KLF4 protein expression presented in **(A)**. **(C)** Western blot image showing the protein expressions of autophagy markers, ATG5, Beclin1, and LC3, in HBMECs from different groups. **(D,E)** Quantification of protein expressions exhibited in **(C)**. **(F)** Western blot showing the protein expressions of ZO-1, occludin, claudin-5, P120, and β-catenin in HBMECs exposed to different conditions. **(G,H)** Quantitative analysis of protein expressions showed in **(F)**. **(I,J)** Representative immunofluorescence stain of LC3 and ZO-1 in HBMECs that suffered from different interventions, respectively. Scale bar = 25 µm. All results are displayed as the mean ± SEM, *n* = 3. *p < 0.05, **p < 0.01, and ***p < 0.001.

PI3K/AKT and FOXO1 have well been proved to be involved in SCI, but whether KLF4 is important for ALG-bFGF hydrogel inhibiting autophagy and maintaining BSCB integrity remains unclear. In the present study, KLF4 inhibitor NSC-664704 was performed in OGD-stimulated HBMECs combined with treatment of ALG-bFGF hydrogel, and then protein levels of autophagy markers and BSCB integrity indicators (AJs and TJs) were determined using Western blot. As shown in Figures 6A,B, ALG-bFGF hydrogel significantly upregulated KLF4 protein expression in OGD-stimulated HBMECs, but this upregulation was dramatically attenuated by NSC-664704, suggesting that ALG-bFGF hydrogel could activate KLF4. In addition, the inhibitory effect of ALG-bFGF hydrogel on autophagy activation in OGD-induced HBMECs was sharply reversed by NSC-664704 (Figures 6C–E), indicating that KLF4 is involved in the role of ALG-bFGF hydrogel inhibiting autophagy. Meanwhile, the role of ALG-bFGF hydrogel in preventing loss of AJ and TJ proteins was also distinctly diminished by NSC-664704 (Figures 6F–H), revealing that ALG-bFGF hydrogel reducing OGD-induced loss of AJ and TJ proteins in HBMECs is regulated by KLF4. Consistent with these results, ALG-bFGF hydrogel-mediated reduction of LC3 fluorescence intensity and enhancement of ZO-1 fluorescence intensity were reversed by NSC-664704 in OGD-stimulated HBMECs, respectively (Figures 6I,J). Taken together, these data provide evidence for the hypothesis that KLF4 might be important for ALG-bFGF hydrogel maintaining BSCB integrity *via* inhibiting autophagy in mice after SCI.

## Discussion

SCI is a devastating neurological disease and BSCB disruption is an important barrier for SCI recovery. bFGF has been proved to possess neuroprotective effects and to promote locomotor functional recovery in SCI *via* various regulatory mechanisms, including reducing BSCB permeability and regulating autophagy. However, the short half-life of bFGF limits its therapeutic efficacy in SCI after systemic or local administration. This study designed an ALG-bFGF hydrogel that could control the release of bFGF to overcome the limitation. The current study demonstrates that *in situ* injection of ALG-bFGF hydrogel obviously inhibits autophagy activation caused by SCI, which contributes to effectively prevent BSCB destruction and thereby significantly improve locomotor function recovery after SCI. Moreover, this study further indicates that the inhibitory effect of ALG-bFGF hydrogel on autophagy is regulated by PI3K/Akt/FOXO1/KLF4 signaling pathway. This study provides a promising strategy using ALG-loaded bFGF to treat SCI.

The sodium alginate–loaded bFGF (ALG-bFGF) hydrogel significantly prevented BSCB leakage and promoted restoration of posterior limb movement after SCI. As a highly biodegradable, biocompatible, and water-soluble substance (Guo et al., 2020), sodium alginate has been widely used as a bioengineering scaffold for tissue regeneration in damaged tissues, including skin (Yanlun Zhu et al., 2020; Shafei et al., 2020), diabetic wounds (Gustinelli Barbosa et al., 2020), bone (Valido et al., 2020), and peripheral nerve system (Szarek et al., 2013; Chaw et al., 2015; Rahmati et al., 2021). Moreover, sodium alginate has potential application in treatment for traumatic brain injury (Kun Zhang et al., 2018). As a biomaterial scaffold, the sodium alginate hydrogel–loaded bFGF showed appropriately porous structures for bFGF loading and release into the injured spinal cord, therefore substantially improving the therapeutic effects of bFGF in SCI.

It is well known that maintaining the integrity of BSCB effectively improves SCI repair after SCI (Lee et al., 2012). BSCB breakdown allows immune cells to infiltrate the damaged area, leading to severe secondary damage (Penas et al., 2007). Furthermore, administration of free bFGF has been shown to protect the integrity of BSCB after SCI (Ye et al., 2016). As key components of BSCB, prevention loss of TJ and AJ proteins may be a potential mechanism for the neuroprotective effect of ALG-bFGF hydrogel in SCI. The data of the present study demonstrated that ALG-bFGF hydrogel treatment significantly improved hind limb movements of mice after SCI, reduced SCI-induced leakage of EB dye in spinal cord, and dramatically prevented decrease of TJ (ZO-1, claudin-5, occludin) and AJ (P120, β-catenin) proteins *in vivo* and *in vitro*. These protective effects of ALG-bFGF hydrogel were significantly better than those of free bFGF treatment, suggesting that ALG-bFGF hydrogel therapy may be a promising therapeutic strategy for SCI.

Autophagy is a highly conserved process for the cellular degradation of long-lived proteins and damaged organelles by the lysosome to maintain cellular homeostasis (Klionsky and Emr, 2000). Complete autophagy contains not only formation of the autophagosome but also autophagosome–lysosome fusion. The progress of the delivery to and fusion of autophagosome with lysosome, and then degradation by lysosomal acid hydrolases is termed autophagy flux. A number of conserved proteins, such as ATG5, Beclin1, and LC3, are required for induction of autophagosome and autophagy flux (Yang et al., 2013). Ample evidence has supported that autophagy is implicated in SCI (He et al., 2016; Zhou et al., 2020). The activation of autophagy may be either induced (Yin et al., 2019; Sipin Zhu et al., 2020) or attenuated (Zhao et al., 2017; Li et al., 2018) after SCI, which depends on the location and severity of traumatic injury, injury model, and species difference. A previous study has shown that excessive autophagy plays a destructive role during SCI, and inhibition of autophagy by free bFGF promotes recovery of motor function in mice (Zhang et al., 2013a). Consistent with this study, our data showed that acute SCI or OGD induced a significant activation of autophagy leading to a sharp reduction of AJ and TJ proteins. However, ALG-bFGF hydrogel could distinctly attenuate the activation of autophagy contributing to increase of AJ and TJ proteins, revealing that ALG-bFGF hydrogel protects the integrity of BSCB by inhibiting autophagy after SCI.

As the primary downstream signal of bFGF, PI3K/Akt has an important neuroprotective role in SCI (Zhang et al., 2013b). Previous studies have confirmed that PI3K/Akt/FOXO1 pathway is involved in SCI (Xu et al., 2021) and can regulate autophagy (Liu et al., 2014). Moreover, KLF4 is proved to be a downstream target of FOXO1 (Huang et al., 2021). Our work further focused on the role of PI3K/Akt/FOXO1/KLF4 signaling pathway by which ALG-bFGF hydrogel protects BSCB integrity *via* attenuation of autophagy after SCI. *In vivo* and *in vitro* investigations demonstrate that administration of ALG-bFGF hydrogel leads to significant activation of AKT, downregulation of FOXO1, and upregulation of KLF4 after SCI or OGD exposure, while these effects were obviously reversed by inhibition of AKT activation with PI3K inhibitor LY294002. Furthermore, the roles of ALG-bFGF hydrogel in attenuating autophagy activation and in protecting BSCB integrity were also significantly overturned by PI3K inhibitor after SCI. Evidence has confirmed that overexpression of KLF4 plays a positive role in SCI (Huang et al., 2020). In our study, KLF4 protein expression was increased by ALG-bFGF hydrogel and downregulated by PI3K inhibitor. To further verify if KLF4 participates in roles of ALG-bFGF hydrogel in inhibiting autophagy and then protecting BSCB, KLF4 inhibitor NSC-664704 was applied in OGD-stimulated HBMECs. Our data demonstrated that both effects of ALG-bFGF hydrogel on inhibiting autophagy activation and reducing loss of BSCB-related proteins were significantly weakened by KLF4 inhibitor. Collectively, our data provide a possibility that PI3K/Akt/FOXO1/KLF4 pathway is involved in effects of ALG-bFGF hydrogel on regulation of autophagy and BSCB permeability.

## Conclusion

**FIGURE 7:**
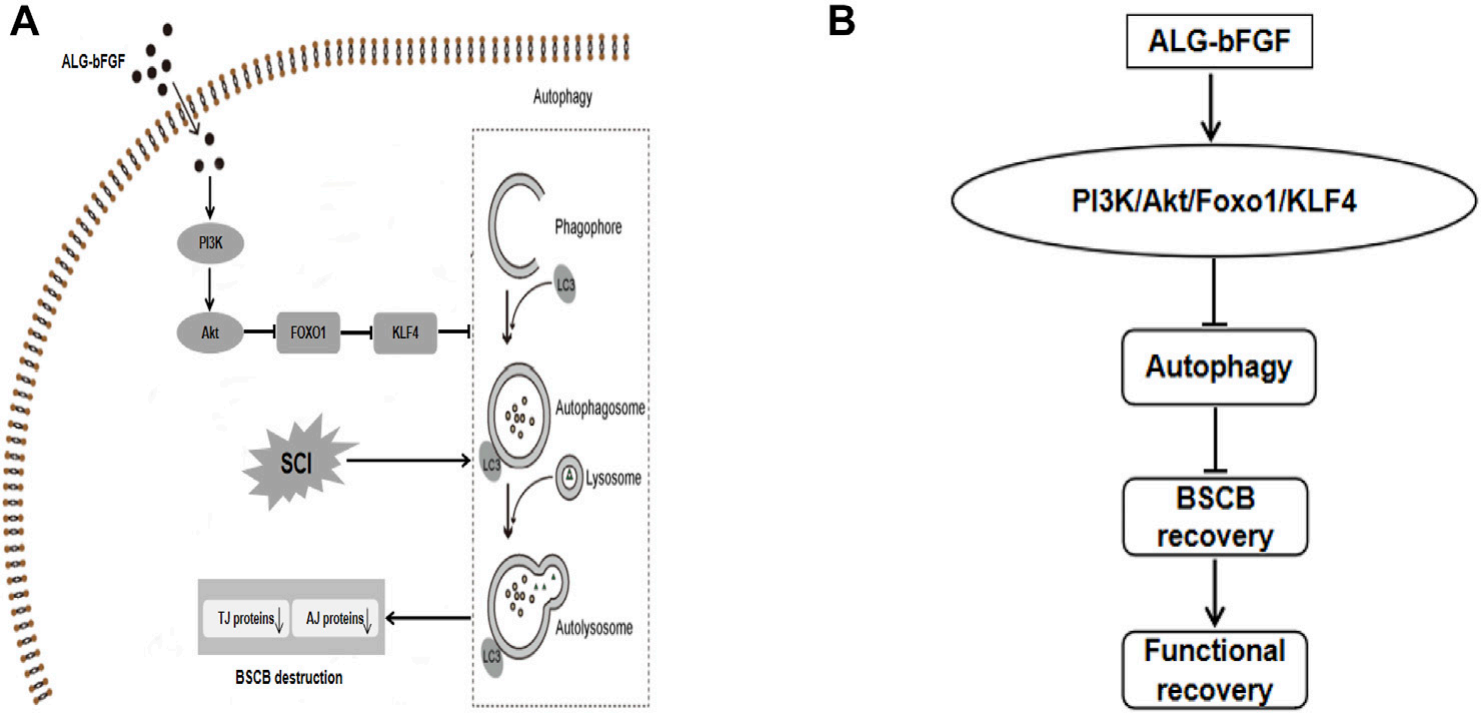
Schematic diagram showing the potential molecular mechanism by which ALG-bFGF hydrogel promotes BSCB recovery after SCI. Acute traumatic SCI induces autophagy activation, which promotes loss of BSCB integrity-related AJ and TJ proteins, leading to limitation on SCI recovery. ALG-bFGF hydrogel improves BSCB repair *via* inhibiting autophagy in a PI3K/Akt/FOXO1/KLF4–dependent manner **(A,B)**.

This study demonstrated that single *in situ* injection of ALG-bFGF hydrogel leading to sustained release of bFGF into damaged spinal cord exhibits more therapeutic effect for SCI. Moreover, the present study confirmed that ALG-bFGF hydrogel improved BSCB repair by inhibition of autophagy activation following SCI. Furthermore, this study revealed that ALG-bFGF hydrogel attenuated autophagy activation contributing to prevention of BSCB destruction by regulation of PI3K/Akt/FOXO1/KLF4 pathway (Figure 7). In summary, our data suggest that ALG-bFGF hydrogel may be a promising therapeutic strategy for traumatic SCI.

## Data Availability

The original contributions presented in the study are included in the article/supplementary material, further inquiries can be directed to the corresponding authors.
